# Predicting Active NBA Players Most Likely to Be Inducted into the Basketball Hall of Famers Using Artificial Neural Networks in Microsoft Excel: Development and Usability Study

**DOI:** 10.3390/ijerph18084256

**Published:** 2021-04-16

**Authors:** Po-Hsin Chou, Tsair-Wei Chien, Ting-Ya Yang, Yu-Tsen Yeh, Willy Chou, Chao-Hung Yeh

**Affiliations:** 1 Department of Orthopedics and Traumatology, Taipei Veterans General Hospital, Taipei 112, Taiwan; choupohsin@gmail.com; 2 School of Medicine, National Yang Ming Chiao Tung University, Taipei 112, Taiwan; 3 Department of Medical Research, Chi-Mei Medical Center, Tainan 700, Taiwan; smile@mail.chimei.org.tw; 4 Medical Education Center, Chi-Mei Medical Center, Tainan 700, Taiwan; u102001309@cmu.edu.tw; 5 School of Medicine, College of Medicine, China Medical University, Taichung 400, Taiwan; 6 Medical School, St. George’s University of London, London SW17 0RE, UK; jess97yeh@gmail.com; 7 Department of Physical Medicine and Rehabilitation, Chi Mei Medical Center, Tainan 700, Taiwan; 8 Department of Neurosurgery, Chi Mei Medical Center, Tainan 700, Taiwan

**Keywords:** Hall of Fame, artificial neural network, convolutional neural network, nurse, Microsoft Excel, receiver operating characteristic curve, Microsoft Excel

## Abstract

The prediction of whether active NBA players can be inducted into the Hall of Fame (HOF) is interesting and important. However, no such research have been published in the literature, particularly using the artificial neural network (ANN) technique. The aim of this study is to build an ANN model with an app for automatic prediction and classification of HOF for NBA players. We downloaded 4728 NBA players’ data of career stats and accolades from the website at basketball-reference.com. The training sample was collected from 85 HOF members and 113 retired Non-HOF players based on completed data and a longer career length (≥15 years). Featured variables were taken from the higher correlation coefficients (<0.1) with HOF and significant deviations apart from the two HOF/Non-HOF groups using logistical regression. Two models (i.e., ANN and convolutional neural network, CNN) were compared in model accuracy (e.g., sensitivity, specificity, area under the receiver operating characteristic curve, AUC). An app predicting HOF was then developed involving the model’s parameters. We observed that (1) 20 feature variables in the ANN model yielded a higher AUC of 0.93 (95% CI 0.93–0.97) based on the 198-case training sample, (2) the ANN performed better than CNN on the accuracy of AUC (= 0.91, 95% CI 0.87–0.95), and (3) an ready and available app for predicting HOF was successfully developed. The 20-variable ANN model with the 53 parameters estimated by the ANN for improving the accuracy of HOF has been developed. The app can help NBA fans to predict their players likely to be inducted into the HOF and is not just limited to the active NBA players.

## 1. Introduction

The Naismith Memorial Basketball Hall of Fame [1] is the highest career honor one can achieve after retirement for a player from National Basketball Association (NBA). When we hear the words “Hall of Fame,” various terms and implications come to our mind, such as greatness, distinction, honor, and various others [2]. Thus, it intrigues authors to expect that those admitted to a Hall of Fame (HOF) should be the epitomes of those traits by observing their NBA career stats and accolades. Not only should HOF be great players, but great honors for active players to pursuit. The premise to be eligible on the HOF ballot is the player who must be fully retired for at least three years [3].

### 1.1. To Know the HOF Probability(or Classification) of an Active NBA Player

Some recently active players are clear locks to be inducted into the HOF well before retirement or their three-year waiting period comes to an end, such as LeBron James and Kevin Durant, who are not yet eligible and inducted at the present time. There are no serious debates about the fate of players of the caliber, but when it comes to other regular players who (1) never quite rose to that level of superstardom and (2) had a doubt about whether they are likely to be inducted into the HOF. We assumed that if the players were to stop playing tomorrow, what are the HOF probability and the classification at present.

What statistics or accomplishments (e.g., accolades) that can help the voters to determinate the HOF inductees seems to be of importance. The basic nomination criterion used for screening out the applicants can adopt a technique called linear logistic regression [4] or nonlinear artificial neural networks (ANN) [5] when a binary label has been identified (e.g., HOF and Non-HOF classified for retired NBA players). The algorithm approach is based on that one or more predictor variables are selected, and the resulting model parameters can be used to classify the HOF or non-HOF category.

When searching the keywords of “NBA and Hall and Fame and basketball” in PubMed library, we have not yet found any article related to the prediction of NBA players inducted into the HOF. Only both websites [6,7] have well-known HOF probability using logistic regression. We were motivated to apply the ANN and convolutional neural network (CNN) for predicting current NBA players inducted into HOF.

### 1.2. ANN and CNN Models Used for Classifying the HOF and Non-HOF

The ANN is a component of artificial intelligence that is meant to simulate a functioning human brain [8]. ANN is the foundation of artificial intelligence (AI) for solving problems that would otherwise be impossible or very difficult by human statistical standards [9]. It was worth using ANN to examine the HOF or non-HOF for active players who are potential to be the inductees of HOF.

The CNN has been applied to many health informatics [10,11,12,13,14]. Its architecture can be described as an interleaved set of feedforward layers implementing convolutional filters followed by reduction, rectification, or pooling layers [15,16,17]. For each layer, the CNN creates a high-level abstract feature. The evidence of the prediction accuracy (up to 7.14%) higher than the traditional linear regression models has been supported [17]. Using either ANN or CNN is a good merit of way for classifying the HOF and non-HOF.

### 1.3. Online Classification Using Smartphones Is Required

As with the advancements in web-based technologies, mobile health communication is rapidly improving [18]. Yet there is no smartphone app designed for classifying HOF. Once the ANN (or CNN) algorithm is applied to estimate the HOF model’s parameters, the HOF classification for a specific NBA player is immediately shown on the smartphone. We are eager to fulfill the gap of lacking the HOF prediction app in the previous studies [6,7].

### 1.4. Objectives

We assumed that if the NBA active players were to stop playing tomorrow, what are the probabilities inducted into the HOF and made a hypothesis of whether an app can be developed for predicting the active NBA players likely to be inducted into the basketball HOF in this study. The following tasks were aimed in three parts:

Part 1: model building includes (1) determining the featured variables used for estimating model parameters and (2) comparing the model accuracies between the two ANN/CNN models.
Part 2: predicting the HOF and developing a HOF app comprise (3) illustrating the most underrated and overrated HOF/Non-HOF players and (4) designing a HOF app.
Part 3: interpreting the resulting HOF/Non-HOF consist of (5) interpreting the reason for HOF or Non-HOF and (6) clustering the active NBA players in characteristics (e.g., features toward stats, accolades, or others).

## 2. Materials and Methods

### 2.1. Data Source

We downloaded 4728 NBA-player career stats and accolades from the NBA website [19] in March 2021. Three categories of NBA players were classified, including (1) the HOF players, (2) the retired Non-HOF players, and (3) the active NBA players.

The training set (including both total in (1) and some in (2) category, respectively) was used to predict the testing sample(involving the remaindings in (2) category and the whole in (3) category). The data are deposited in Appendix A.

All downloaded data have met the requirement for analyzing information from public websites. Ethical approval is not necessary for this study because neither human subjects nor personal data were accessed.

### 2.2. Concept in Model Building and Parameter Estimation

A Microsoft Excel visual basic application module was used to handle the data. Three parts are involved in this section.

#### 2.2.1. Featured Variables Extracted from the NBA Stats and Accolades

Featured variables were extracted from the NBA-players’ stats, and accolades in the website [19] based on the criterion of no missing data in the variables through following two steps:

Step 1: The correlation coefficients (CC) between HOF and the variables must be beyond 0.10.

Step 2: The variables with higher CCs(>0.10) were put into logistical regression analysis. The significant level of α error (i.e., the type I error) was set at 0.05. The featured variabes was displayed on a forest plot [20,21].

#### 2.2.2. Model Building and Parameter Estimation

After variables selected (i.e., Type I error less than 0.05) in 2.2.1, the ANN and the CNN models were built for comparing their model accuracies (e.g., sensitivity(SENS), specificity(SPEC), and area under the receiver operating characteristic curve (AUC) [22,23].

**Figure 1:**
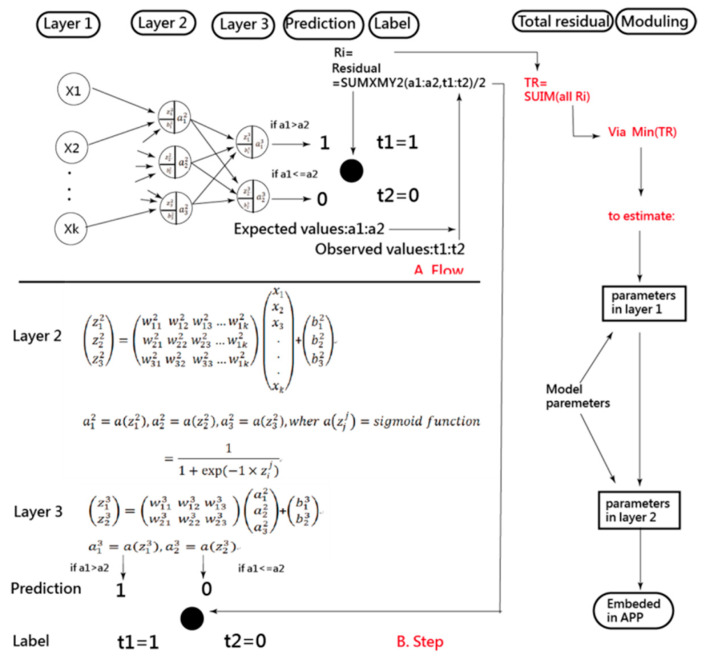
The process of estimating parameters in the artificial neural network (ANN) model.

The CNN performed on Microsoft Excel has been illustrated before [12,13,14] while ANN has not been paired along with CNN in MS Excel. As demonstrated in Figure 1, the ANN process involves data input in layer 1 where the data are joined with two types of parameters and run through the sigmoid function algorithms in layers 2 and 3. Finally, as shown on the right side and the bottom of Figure 1, the predictive model was optimized when the total residuals were minimized through the MS Excel function of sumxmy2 and solver add-in; see the two modules in Appendix A and Appendix B.

#### 2.2.3. Comparion of Model Accuracy between the two ANN/CNN Models

The training set was collected from the 85 HOF and 113 retired Non-HOF players based on (1) their completed data(e.g., some career stats missed in the early NBA(or ABA) stage, e.g., STL, BLK, TOV, PF, PTS, 3P, 3PA, 3P%, 2P, 2PA, 2P%, and eFG%) and (2) a longer career length (≥15 years (i.e., a few HOF players have shorter career length, e.g., Dino Radja and Dražen Petrović with only 4-year NBA experiences).

The balanced-class data were another important issue that should be considered. Otherwise, the imbalanced-class data [24,25] lead to an extremely imbalanced ratio (= SENS/SPEC or SPEC/SENS) while the modle pursuits the ultimate accurate rate of prediction (i.e., by minimizing the residuals). In this study. The ratio of the class number in the training set is 0.75 (= 85/113 > 0.5).

### 2.3. Tasks in Achieving the Study Goals

#### 2.3.1. Model Buiding and Model Comparison

Task 1: Selection of Featured Variables

The featured variables were displayed on a forest plot [20,21].

Task 2: Comparison of Accuracies between the Two ANN and CNN Models

The accuracy was determined by observing the higher SENS, SPEC, precision, F1 score, accuracy, and AUC in both models. The definitions are listed below:

(1) True positive (TP) = the number of predicted NIQJ to the true NIQJ,
(2) True negative (TN) = the number of predicted Non-NIQJ to the true Non-NIQJ,
(3) False-positive (FP) = the number of Non-NIQJ minuses TN,
(4) False-negative (FN) = the number of NIQJ minuses TP,
(5) SENS = Sensitivity = true positive rate (TPR) = TP ÷ (TP + FN),
(6) SPEC = Specificity = true negative rate (TNR) = TN ÷ (TN + FP),
(7) Precision = positive predictive value (PPV) = TP ÷ (TP + FP),
(8) F1 score = 2 × PPV × TPR ÷ (PPV + TPR),
(9) ACC = accuracy = (TP + TN) ÷ N,
(10) N = TP + TN + FP + FN,
(11) AUC = (1 − Specificity) × Sensitivity ÷ 2 + (Sensitivity + 1) × Specificity ÷ 2,
(12) SE for AUC = √(AUC × (1-AUC) ÷ N),
(13) 95%CI = AUC ± 1.96 × SE for AUC,

The generalization capability was determined by model accuracy using the training sample to predict the testing sample.

#### 2.3.2. HOF Prediction and APP Development

Task 3: Unexpected Classifications of HOF for NBA Players

In the training sample, the unexpected classification cases appear in two underrated and overrated situations.

Two scenarios exist in the training sample: (1) the overrated HOF players and (2) the underrated Non-HOF retired players.

One scenario in the testing retired sample: the underrated players who have not yet been inducted into the HOF.

One scenario in the active player sample: the potential players who are likely nominated to the HOF. We assumed if they are to stop playing tomorrow. A visual representation would be used to interpret the surprisingly unexpected cases in Results.

Task 4: An App Developed for Predicting HOF

A HOF app was designed to classify the HOF/Non-HOF groups using the ANN(or CNN) model. The classification will be shown on smartphones. A dashboard displayed on Google Maps was designed for the binary (HOF and Non-HOF) category probabilities plotted by the Rasch category characteristic curve [26,27].

#### 2.3.3. Data Interpretations and the Characteristics of Active NBA Players

Task 5: A Visual Display to Interpret the Reason for HOF or Non-HOF

In order to examine the deviation from the average career stats and accolades yielded from the 85 HOF players, an individual player was compared across all featured variables shown in a firest plot.

Task 6: Using Social Network Analysis to Cluster the Active NBA players

Social network analysis(SNA) [28,29] was performed to cluster active NBA players by observing their co-occurrence events, including featured variables and player names; see the MP4 video in Appendix B.

Similar to the exploratory factor analysis on questions or items in a survey, the cluster analysis of SNA was performed to examine (2) how many clusters in active NBA players and (2) which features or characteristics can be appropriately named for each cluster. In a visual representation, bubbles are colored and sized by clusters and the centrality degrees of each entity(i.e., player and variable). The bigger bubble stands for a higher probability of being inducted into the HOF.

### 2.4. Statistical Tools and Data Analysis

IBM SPSS Statistics 22.0 for Windows (SPSS Inc, Chicago, IL, USA) and MedCalc 9.5.0.0 for Windows (MedCalc Software, Ostend, Belgium) were used to perform the descriptive statistics, frequency distributions among groups, logistic regression analyses, and the computation of model prediction indicators mentioned in Eqs from 1 to 13. The significant level of type I error was set at 0.05. Both ANN and CNN were performed on MS Excel (Microsoft Corp); see Appendix A.

**Figure 2:**
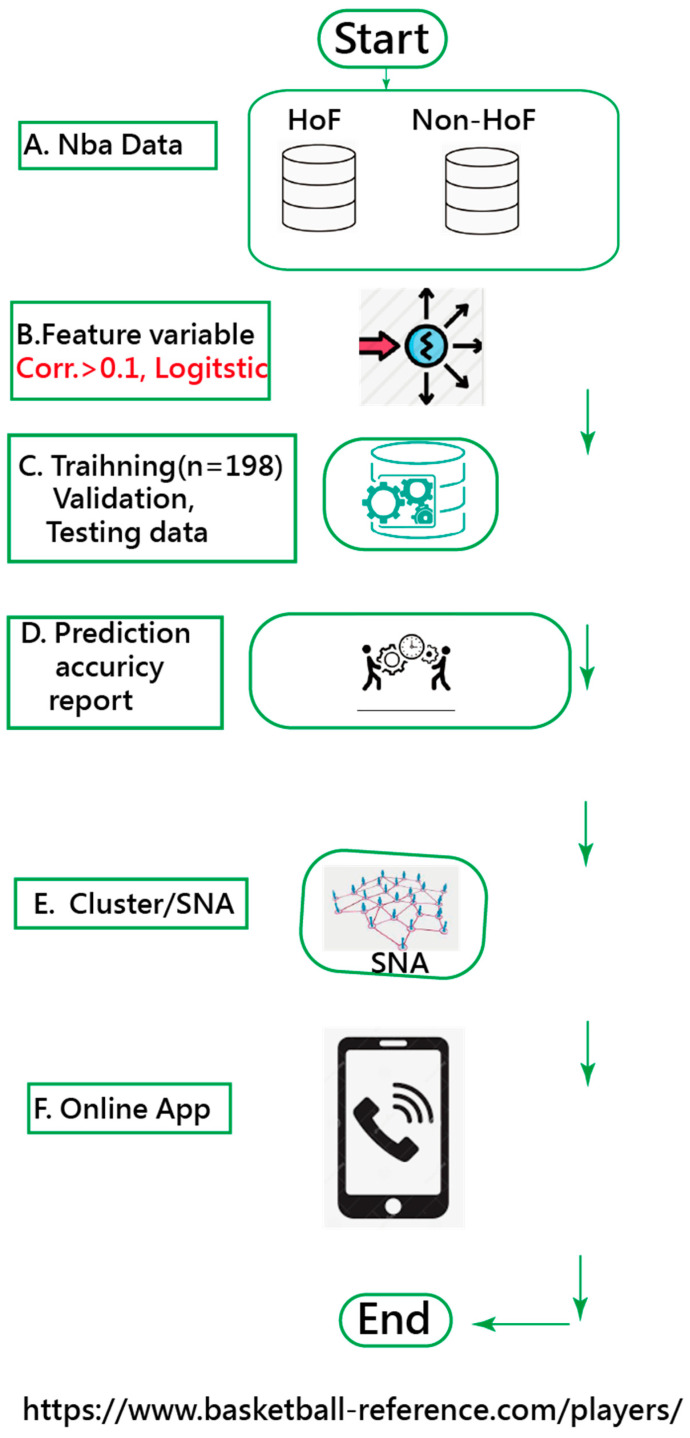
The study flowchart.

A visual representation of the classification was plotted using two curves based on the Rasch model [26,27]. The study flowchart and the ANN modeling process are shown in Figure 2 and Appendix B with an MP4 video.

## 3. Results

### 3.1. Descriptive Statistics

All those 4728 NBA players were split into three parts: (1) the 152 HOF members, (2) the 4173 retired Non-HOF, and (3) the 707 active NBA players. The training sample consists of 198 members (85 and 113 for HOF and Non-HOF) shown in Table 1. Only 3.11 percent of the NBA players were possiblely inducted into the Hall of Fame (see the first row in Table 1).

In the 4173 retired NBA players, shoots with the left hands account for 6% compared to the 94% of them using right hands. The HOF players have a longer mean career length (= 12.1, SD = 4.0) than the Non-HOF (= 4.7, SD = 4.3). The two HOF/Non-HOF samples have equivalently equal body heights and weights. The HOF players have more accolades received in their NBA career than the Non-HOF players in the 4173 sample.

### 3.2. Model Buiding and Model Comparison

#### 3.2.1. Task 1: Selection of Featured Variables

**Figure 3:**
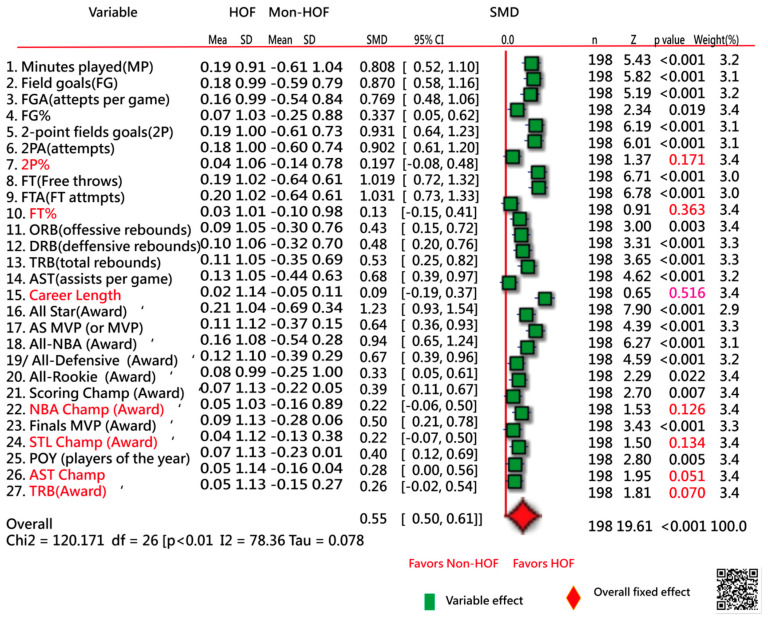
Eligible featured variables extracted from the 27 variables based on the correlation coefficient (>0.1) associated with the Hall of Fame (HOF) label.

Figure 3 [30] shows the 27 variables in comparisons to the standardized mean difference (SMD) between the two HOF/Non-HOF samples in the 198-player training set. No difference was found in the seven variables that were excluded from this study. The remaining 20 featured variables were used for estimating model parameters and predicting the HOF probability for each NBA player.

#### 3.2.2. Task 2: Comparison of Accuracies between the Two ANN and CNN Models

Table 2 shows a higher AUC of 0.93 (95% CI 0.93–0.97) in classification under the ANN model using the 20 feature variables extracted from Figure 3. The ANN performed slightly better than the CNN on the classification accuracy of AUC (= 0.91, 95% CI 0.87–0.95).

### 3.3. HOF Prediction and APP Development

#### 3.3.1. Task 3: Unexpected Classifications of HOF in NBA Players

**Figure 4:**
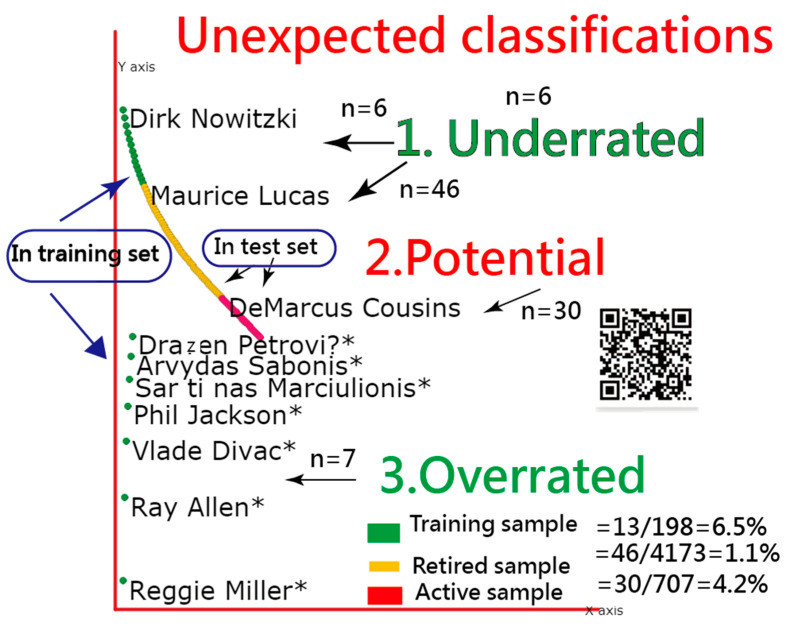
Unexpected findings of HOF players with underrated and overrated expectations (note: * denotes the player has been inducted into HOF).

The training sample has a slightly higher misclassification rate (= 6.5% = 13/198) than the other two testing samples of 1.2% and 4.2% (i.e., for the remainding retired and the active NBA players); see Figure 4.

In the 85 HOF players under the ANN model, we found that seven HOF inductees with red bubbles in Figure 4 are overrated according to their career stats and awards. They are Phil Jackson, ReggieMiller, and Ray Allen, and the other three European NBA players due to less number of accolades in their NBA career.

As for the retired players in the training set, six with green bubbles in Figure 4 are underrated, including Dirk Nowitzki, Pau Gasol, Tom Chambers, Dwyane Wade, Walter Davis, and Chris Webber. They are eligible to be inducted into the HOF according to our 20 featured variables. The reason for explaining the cause might be other special credits or achievements in their NBA career; see the training sample shown in the link [31].

The third part is regrading the retired players with yellow bubbles in Figure 4. A total of 46 players are underrated, such as Amar’e Stoudemire, Stephon Marbury, Carlos Boozer, Chris Bosh, Tim Hardaway.

The fourth part is the 30 active NBA players who have potential to be inducted into the HOF, sucha as LeBron James, Chris Paul, Kevin Durant, Russell Westbrook, James Harden, Stephen Curry, Dwight Howard, Carmelo Anthony, Anthony Davis, Damian Lillard, Paul George, Kyrie Irving, Blake Griffin, Kawhi Leonard; see the active NBA players shown in the link [32].

Readers are also invited to scan the QR-code in Figure 4 (or via the link [33]) and click on the bubble of interest. The player’s profile would immediately appear on the website [19].

#### 3.3.2. Task 4: An App Developed for Predicting HOF

**Figure 5:**
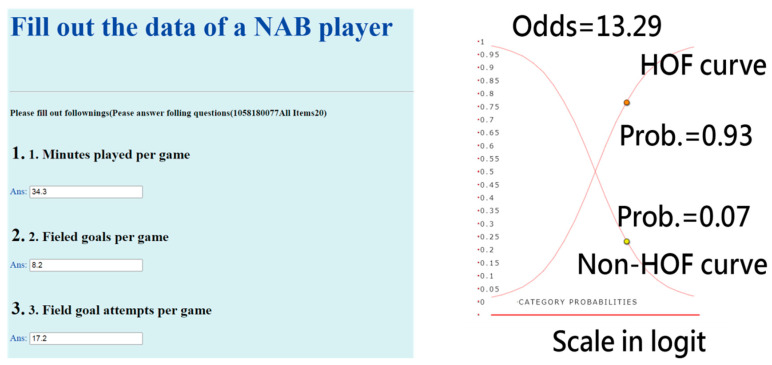
Snapshot of the NIQJ app on a smartphone.

The interface of the APP targeting one NBA player for predicting the HOF is shown on the left-hand side of Figure 5. Readers are invited to click on the links [34,35] and interact with the HOF app, see Appendix B. It is worth noting that all 53 model parameters are embedded in the 20-item ANN model. Once data are submitted, it generates a result as a classification of either HOF or Non-HOF on the smartphone.

An example of Stephen Curry is shown on the right-hand side of Figure 5, from which we can see the high probability (0.93) of HOF, which is the curve starting from the bottom left to the top right-side corner. The sum of probabilities for HOF and Non-HOF is 1.0. The odds can be calculated with the formula (*p*/[1–*p*] = 0.93/0.07 = 13.29), suggesting that Stephen Curry has an extremely high probability(0.93) to be inducted into the HOF based on the recent information.

### 3.4. Data Interpretations and the Characteristics of Active NBA Players

#### 3.4.1. Task 5: A Visual Display to Interpret the Reason for HOF or Non-HOF

**Figure 6:**
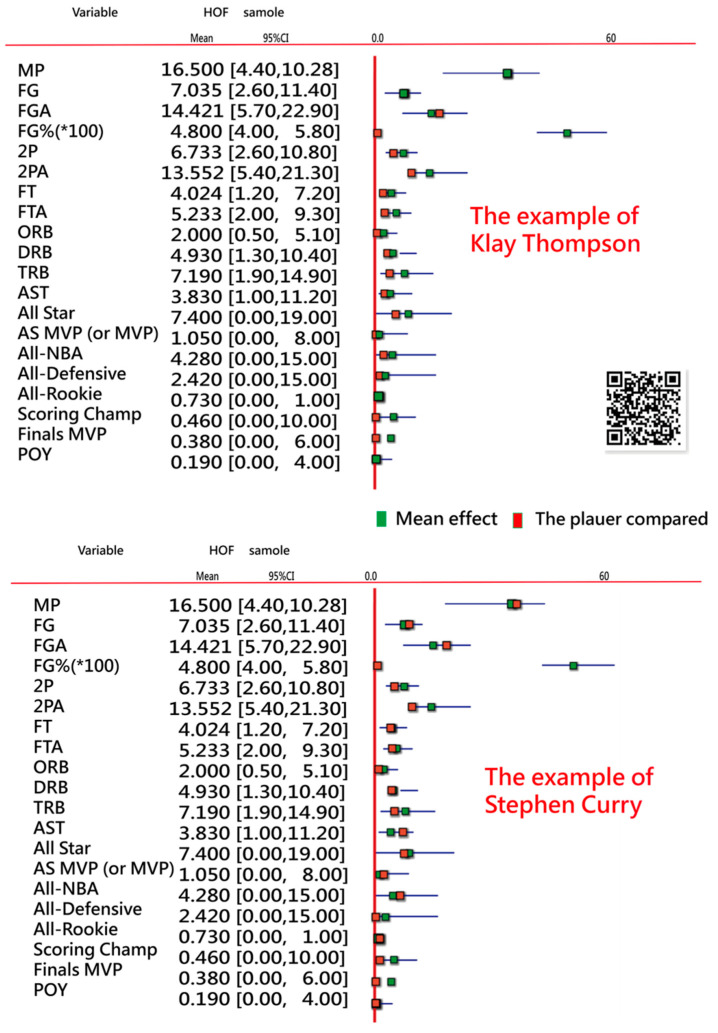
The interpretation of the records compared with the HOF sample for the NBA player Klay Thompson.

The example of Klay Thompson is shown at the top panel in Figure 6 when the NBA career stats and accolades are input into the website [36]. We can see that most data are lower than the mean in the HOF sample. The main reason is the absence of 3P-related variables omc;ied in the featured variables. Readers are invited to input the data of anyone player shown on the website and examine the comparison of the results compared with the 85-HOF-play sample. Klay Thompson is not quantified to be inducted into the HOF at present.

For example, if the career stats and accolades of Stephen Curry are put into the link [36], we can see the bottom panel in Figure 6, more numbers of variables (e.g., AST, MVP, All-NBA, and FGA) are higher than the mean of 85 HOF players in the training sample. As such, Stephen Curry deserves the HOF at present.

#### 3.4.2. Task 6: Using Social Network Analysis to Classify Active NBA Players

**Figure 7:**
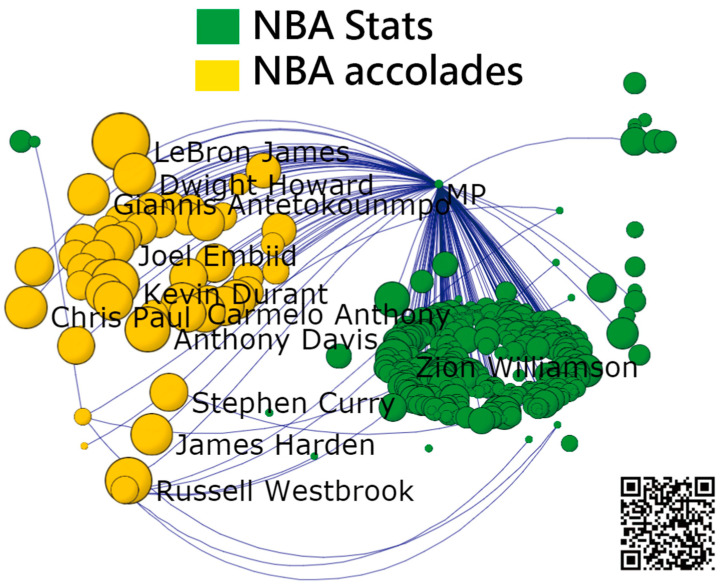
Classification of active NBA players using social network analysis.

The characteristic clusters were analyzed using the SNA. We can see that two clusters (e.g., career stats in green and accolades in yellow) appear in Figure 7 [37]. Most potential active NBA players are clusterd in yellow. The typical representatives in green are Zion Williamson(1st pick drafted by New Orleans Pelicans in 2020) and Trae Young(5th pick drafted by Dallas Mavericks in 2018, now plays for Atlanta Hawks, NBA Draft, 1st overall) who are young without adequet accolades but with exceptioanal stats. Nonetheless, Zion Williamson is classified into the HOF. Trae Young is still classified to the Non-HOF.

In Figure 7, it can be seen that Zion Williamson is attributed to the NBA stats instead of a large number of awards or accolades. Readers are suggested to click on the link [37] for examining the detail about personal information on the dashboard.

### 3.5. Online Dashboards Shown on Google Maps

There are eight links [30,31,32,33,34,35,36,37] provided to readers who can practice the dashboards on their own.

## 4. Discussion

We assumed that if this player retired today, what is the probability he would be elected to the Hall of Fame. There are three parts in the study:

Part 1 (model building and parameter estimation), Part 2 (predicting HOF probability and designing a HOF app), and Part 3 (interpreting resulting HOF/Non-HOF and clustering active NBA players).

A total of six tasks were implemented and found that (1) 20 feature variables in the ANN model yielded a higher AUC of 0.93 (95% CI 0.93–0.97) based on the 198-case training sample, (2) the ANN performed better than CNN on the accuracy of AUC (= 0.91, 95% CI 0.87–0.95), and (3) a ready and available app for predicting HOF was successfully developed.

In addition, the two visualizations (i.e., (1) personal career stats and accolades compared with the mean of HOF players in Figure 6 and feature clusters separated by SNA in Figure 7) are modern and innovative, which were never seen before in the literature.

The hypothesis of whether an app can be used for predicting active NBA players likely to be inducted into the basketball HOF has been supported by the findings in this study.

### 4.1. What This Knowledge Adds to What We Already Knew

Which active NBA players are surefire inductees needs more research and intrigues more NBA fans. Looking at which current players are crafting a HOF resume, some players’ outlooks are clear, while others need more time in determining their likelihood to be inducted.

The top 8 active NBA players currently rank in the HOF candidacy are listed [38] as “Tier 1: First Ballot Inductees” in no particular order: LeBron James, Chris Paul, Kevin Durant, Russell Westbrook, James Harden, Stephen Curry, Dwight Howard, and Carmelo Anthony. All of which (shown in the link [32]) are predicted in our ANN predictive model.

The next eight active NBA players (e.g., Derrick Rose, Giannis Antetokounmpo, Damian Lillard, Paul George, Kyrie Irving, Blake Griffin, Kawhi Leonard, and Anthony Davis) as “Tier 2: Players on Track” were also listed [38] and predicted in the current study [32]. However, the three (Kyle Lowry, Kevin Love, Klay Thompson) were projected on Non-HOF in our study. The reasons for Klay Thompson was given in Figure 6. Readers are invited to click on the link [36] to examine the reasons for Kyle Lowry and Kevin Love, or see the results in Appendix A.

The last 4 active NBA players (e.g., Jimmy Butler, Rajon Rondo, Draymond Green, and John Wall) as “Tier 3: On the Skirts” were identified by Swinton [38]. When referring to our study [32], Draymond Green is not quantified for the HOF now. Although he is a three-time NBA champion, the NBA champion was excluded from the featured variables (*p* = 0.126 in Figure 3). Draymond Green has shown his tenacity on defense, but there is room for him to grow outside the benefits of having Curry and Thompson on the floor [38]. Jimmy Butler is another defensively minded player, but the NBA career stats are better than Draymond Green as of 11 March 2021. The reasons can be examined at our link [36] or referred to Appendix A.

Unfortunately, Swinton [38] had not provided detailed data and HOF calculation/selection for the active NBA players. Bailey [39] utilized players’ averages for career win shares (WS) [40] per 48 min (= WS ÷ MP × 48, where MP is the minutes per game). All those top 25 active NBA players [30], but the three(i.e., Joe Johnson, Kyle Lowry, and Klay Thompson), are consistent with our study [32]. It is worth noting that only the MP (= 34.7 in career stats of Joe Johnson) is higher than the mean MP in the 85 HOF sample (= 33.38).

When our resulting HOF in active NBA players [32] is compared with the results using logistic regression [6], in 30 players, only the six (e.g., Kevin Love, Klay Thompson, Manu Ginobili, Paul Pierce, Tony Parker, and Vince Carter) [6] are inconsistent with our findings. The reasons might be NBA champions excluded from the featured variables in our study. If the comparisons of all featured variables are made for these six players, all those stats and accolades of thesix players are beneath the mean in the 85 HOF samples; see Appendix A or click on the link [36].

Similarly, the difference was also found in another study using logistic regression [7,41] with an example of Tony Parker. His probability is 93.8% for being elected to the HOF. The reasons might be the featured variables are differently selected in both predictive models. For instance, the five criteria of Height (in.), NBA Championships, NBA Leaderboard Points, NBA Peak Win Shares, and All-Star Game Selections were involved in the model [7] instead of the 20 variables used in our model (Figure 3).

Three retired NBA players have been nominated for the 2021 HOF Election [42]. Only Chris Bosh (two-time NBA champion and 11-time NBA All-Star) was successfully predicted in our HOF model. Michael Cooper (five-time NBA champion and five-time NBA All-Defensive First Team) and Paul Pierce (NBA Finals MVP and 10-time NBA All-Star) were deemed as Non-HOF in this study. The reasons might be their NBA career stats relatively lower than the average of the 85 HOF sample.

If previous finalists (2020) were included again this year (2021) for consideration, only Chris Webber (five-time NBA All-Star) was predicted as HOF. The other two Tim Hardaway (five-time NBA All-Star Tim) and Ben Wallace(four-time NBA Defensive Player of the Year) were also excluded from the HOF prediction in this year.

The top 10 active NBA players who are already Hall of Fame worthy were selected [43]. Only Tony Parker was inconsistent with our prediction of HOF. The ten active NBA players who are Hall of Fame locks [44] are 100% consistent with our findings.

In comparison to the 250 players (97 HOF vs. 153 Non-HOF) based on their NBA career leaders and records for Win Shares [45], only 28.67% (= 43/150) met the potential HOF in our prediction. If the variable of Win Shares [40] was included in our ANN model, the accurate rate would be increased. The problem is that no such career win shares were provided on the website [19].

### 4.2. What This Study Contributes to Current Knowledge

We proved the HOF app to readers (or NBA fans) who can upload career stats/accolades of interested NBA players onto the website [34] to (1) examine the HOF classification and (2) compare the differences in featured variables with the mean of the HOF sample. It is worth mentioning that the MVP awards(#17 in Figure 3) have the total number from both Aall-Star MVP and NBA MVP. All career stats and accolades publicly come from the NBA website [19]. That is, no advanced stats and metrics (e.g., Win Shares [40,46]) are required in the HOF ANN model. Those jargon terms might be a bit intimidating because they are unfamiliar and complicated to general NBA fans. No stat is worthless if we know how to use it [46](see next section of strenghts in this study).

### 4.3. Strengths of This Study

We have not yet found any article related to the topic of the most likely HOF player on every current NBA roster till now. Although numerous websites [38,39,41,42,43,44,45] reported the ten active NBA players who are Hall of Fame locks. None of them provided such a thing as the perfect application of a statistic to readers, but from the human aspect and by their own subjective judgments [46]. The two websites [6,7] applied logistic analysis to make NBA Hall of Fame Probability. However, no accuracies (e.g., SENS, SPEC, and AUC) were provided to readers.

There are three strengths in this study:

1. The first peer-review study applied the ANN and the CNN to predict the active NBA players inducted into the HOF. The evidence shows the prediction accuracy (up to 7.14%) higher than the traditional linear regression models [17].
2. The study was conducted under Microsoft Excel that is familiar to ordinary readers who can replicate the study on their own with MP4 video, ANN/CNN modules, and the original data are provided in Appendix A and Appendix B.
3. All visual dashboards on Google Maps are advanced and novel, never seen before in NBA communities, such as those websites [6,7,38,39,41,42,43,44,45].

### 4.4. Implications of the Results and Suggested Actions

ANN was performed on MS Excel, which has not been reported in the literature. An app was designed to display the HOF classification results using the categorical probability theory in the Rasch model [26,27]. The animation-type dashboard was incorporated in the ANN model to enable easy understanding of the classification results with visual representations.

Many different types of algorithms for classification in machine learning [47,48] were applied in the literature, such as logistic regression, support vector machine [48], naïve Bayes, random forest classification, ANN, CNN [12,13,14], and k-nearest neighbor [48]. ANN was deemed to be superior to the other algorithms, with 93.2% classification accuracy in a previous study [47]. It is equivalently equal to 0.93 (95% CI 0.93–0.97) in this study and worth further comparing the ANN accuracy in the future.

We built an app to display the HOF/Non-HOF classifications using the visual dashboard on Google Maps. The animation-type dashboard was incorporated in the ANN model to enable readers to (1) understand the classification results with visual representations and (2) practice it on their own with the links [34,35], which has not been reported in the literature, particularly using forest plots to display feature variables in comparison. As a result, the app enables us to continuously monitor the active NBA players who are Hall of Fame locks or still on the bubbles on the bubble [44].

Furthermore, we applied the minimization of average model residuals in both classes to obtain balanced HOF and Non-HOF and overcome the disadvantage of the imbalanced SENS and SPEC, albeit with high accuracy rates. It is of importance to examine the balanced SENS and SPEC instead of the high accuracy rates only.

The categorical probability curves are shown in Figure 5. The binary categories (e.g., success and failure of an assessment in the psychometric field) have been frequently applied in health-related outcomes [12,13,14,49,50,51]. However, we are the first to provide the categorical probability curves of the HOF animation-type dashboard displayed on Google Maps (Figure 5).

### 4.5. Limitations

There are several limitations to this study. First, caution should be taken when interpreting and generalizing findings beyond the featured variables applied in the ANN models. For instance, the number of NBA champions was excluded from this study because of the weak discrimination between HOF and Non-HOF(*p* = 0.126 in Figure 3). As such, Tony Parker, with 4-time NBA champions, is not outstanding when compared to others with at least 4-time NBA champions [e.g., Jim Loscutoff(7 times), Robert Horry(7), Larry Siegfried(5), Derek Fisher(5), Ron Harper(5), Steve Kerr(5), Michael Cooper(5), Gene Guarilia(4), Will Perdue(4), Manu Ginóbili(4), Pep Saul(4), Tony Parker(4), Kurt Rambis(4), LeBron James(4), and Horace Grant(4), and John Salley(4)]

Second, we have not discussed possible further improvement in predictive accuracy. For instance, whether other featured variables (e.g., variables are not included in Figure 3) applied to the ANN model can increase the accuracy rate is worth discussion. It would be useful in the future to look for other variables that can improve the power of the model prediction.

Third, the study was carried out on the ANN and CNN models/ whether other predictive models have higher accuracy and stability than the ANN has yet to be investigated.

Fourth, the eligible players on the ballot for HOF honors are not possibly 100% determined by the predictive model. A special case and eligibility are reviewed on an individual basis. Remember, the Hall is not based solely on NBA. For example, a shorter NBA career length is impossible to have a huger number of accolades or NBA awards, but they [e.g., Buddy Jeannette (3 years), Alfred McGuire(3), Maurice Stokes(3), and John Thompson(2)] have been inducted into the HOF.

Finally, we assumed that if the players were to stop playing tomorrow, what are the HOF probability and classification at present. The day of tomorrow is just subject to the data collected on 10 March 2021 from the website [19]. The personal career stats and accolades would be changed (i.e., flucated for career statsand increased for accolades) as the time elapsed.

## 5. Conclusions

The 20-variable ANN model with the 53 parameters estimated by the ANN for improving the accuracy of HOF has been developed with the use of Excel (Microsoft Corp). The app can help NBA fans to predict their players likely to be inducted into the HOF and is not just limited to the active NBA players.

## Figures and Tables

**Table 1:** Comparison of demographic data of the study samples.

Variable	Non-HOF	HOF	*n*	%
**A: All downloaded data**	4728	152	4880	3.11
Testing retired player	4021	152	4173	3.64
Training sample	113	85	198	42.93
* B: Shoots				
Left hand	237	14	251	6
Right hand	3784	138	3922	94
* **C: Career length**				
Mean	4.7	12.2		
Standard deviation(SD)	4.3	4.0		
* D: Body				
Height(cm)	197.9	198.8		
Weight(kg)	93.9	94.0		
* E: Award				
All star	0.18	6.34		
All NBA MVP	0.04	4.40		
All-Defensive	0.05	1.59		
All-Rookie	0.08	0.46		
Scoring Champ	0.00	0.39		
NBA Champ	0.15	1.57		
Finals MVP	0.00	0.24		
BLK(blocks)	0.01	0.09		
TRB(total rebounds)	0.00	0.32		
Sixth Man	0.00	0.01		
AST Champ	0.00	0.32		
POY(play of the year)	0.00	0.11		
STL Champ	0.01	0.09		

Note: * *n* = 4173.

**Table 2:** Comparison of statistics in models and scenarios.

Model	*n*	SENS	SPEC	Precision	F1 Score	ACC	AUC	95%CI
ANN								
Training set	198	0.92	0.95	0.93	0.92	0.93	0.93	0.90–0.97
Testing retired	3975		0.99			0.99		
Testing active	707		0.96			0.96		
CNN								
Training set	198	0.91	0.91	0.93	0.92	0.91	0.91	0.87–0.95

## Data Availability

All data were deposited in Appendix A and Appendix B.

## Appendix A

https://osf.io/xbqc5/?view_only=6752a813740d44e3bd230d4709cb9c79 (accessed on 10 March 2021).

## Appendix B

https://osf.io/5hzrt/ (accessed on 10 March 2021).
